# Consistent Eye Movement Patterns in Static and Dynamic Face Recognition: A Hidden Markov Model Study

**DOI:** 10.3390/brainsci15111173

**Published:** 2025-10-30

**Authors:** Rachel J. Bennetts, Natalie Butcher, Karen Lander

**Affiliations:** 1 Department of Psychology, Brunel University of London, London UB8 3PH, UK; 2 Department of Psychology, Teesside University, Middlesbrough TS1 3BX, UK; n.butcher@tees.ac.uk; 3 Division of Psychology, Communication and Human Neuroscience, University of Manchester, Manchester M13 9PL, UK; karen.lander@manchester.ac.uk

**Keywords:** face recognition, eye tracking, eye movements, hidden Markov models, movement advantage, motion

## Abstract

**Background/Objectives:** Eye movements provide important insights into face processing. Hidden Markov models of eye movements (EMHMMs) are a relatively new approach that identifies common patterns across observers, moving beyond region-of-interest analyses. Prior EMHMM studies with static faces have typically revealed two strategies: a central “holistic” style and a feature-based “analytical” style. However, it is unknown whether such patterns extend to dynamic faces, which more closely reflect real-world viewing. This study is the first to apply EMHMMs to dynamic face recognition. **Methods:** Participants completed a face learning task in which half of the identities were presented as static images and half as dynamic videos. Eye movements were analysed using EMHMMs during both learning and recognition phases. **Results:** Two consistent patterns emerged across conditions: Central-focused and Eye-focused. A small subgroup displayed a third, central-plus-right-eye pattern when learning static faces. Eye movement patterns were largely stable across static and dynamic conditions, with only 16–27% of participants switching between them. Patterns were generally unrelated to recognition accuracy; however, participants adopting Eye-focused patterns during static learning performed better on static recognition. **Conclusions:** EMHMM-identified patterns generalise from static to dynamic faces, indicating strong stability in face-viewing behaviour across stimulus types. This finding contrasts with previous region-of-interest analyses suggesting greater differences between static and dynamic faces. By extending EMHMMs to dynamic faces, this study underscores the value of diverse analytical approaches for capturing eye movement behaviour and advancing understanding in face processing.

## 1. Introduction

Eye movements are a rich source of information about how we process the visual world, and particularly the faces of those around us. A substantial body of work has examined where people look when learning and recognising faces; this work has typically reported a strong focus on the internal features of the face, especially the eye and nose regions [1,2,3,4,5,6], which are considered to contain the most useful information for recognition [4,5]. Although these findings advance our understanding of general face recognition strategies, the role of individual differences in eye movements in shaping recognition performance and the effect of facial movement on eye movement patterns is still unclear.

Recent work has identified substantial variation in eye movement patterns to faces between individuals, e.g., [7,8,9,10,11]. Findings have demonstrated significant between-person variation in scan paths, fixation distributions, and saccade dynamics (for example, see [8]). Moreover, studies have shown that individuals tend to exhibit relatively consistent eye movement patterns over time, even across different face stimuli and tasks [12]. In some instances, eye movement patterns have been linked to face recognition ability [11,13]. For instance, Bobak et al. [13] found that individuals with more severe prosopagnosia (a condition characterised by problems with face recognition) spent less time examining internal face features. Further, some prosopagnosics spent less time examining the eyes and more time examining the mouth than controls. In contrast, individuals who excel at face recognition (“super-recognisers”) may make longer fixations to the inner features of the face (i.e., nose and eyes) [13,14].

More recent work has employed a novel approach to looking at individual differences in eye movement patterns when completing face recognition tasks using eye movement hidden Markov models (EMHMMs e.g., [15,16,17]). Unlike earlier work, which examined how much time participants spent looking at specific pre-defined areas of an image (regions of interest, ROIs; e.g., [9,13]) or focused purely on the spatial distribution of fixations [18,19], EMHMMs use a data-driven approach to derive person-specific ROIs and to cluster participants into groups based on both the spatial distribution of these ROIs, sometimes including their durations, (e.g., [16]), and the pattern of transitions between them [15]. This allows researchers to identify qualitative differences in eye movements between individuals or conditions (by classifying participants into different groups or clusters) as well as quantitative differences between individuals or conditions (by examining how closely eye movement patterns resemble the group pattern). As such, it is well-suited to examining individual differences in face processing. Using this approach, several studies have identified two or three distinct patterns of eye movements when learning and recognising faces. Broadly, these patterns have been conceptualised as reflecting more “holistic” or more “analytic” eye movement patterns, although more recent work often labels these eye movement patterns as “eye-focused” and “nose-focused” [17,20,21], reflecting some level of uncertainty around the actual perceptual strategies underpinning these eye movement patterns. In holistic/nose-focused eye movement patterns, most fixations begin and remain focused on the centre of the face; whereas in analytic/eye-focused eye movement patterns, fixations generally transition from the centre of the face to the eye region [15,16,22,23]. Several papers have found that analytic/eye-focused eye movement patterns are associated with better face recognition ability [16,22,23], e.g., [15,17], which aligns with other eye-tracking work suggesting that the eye region is important for face recognition [9].

The relationship between the analytic/eye-focused eye movement patterns and face recognition accuracy is noteworthy because faces have a limited number of features to focus on (typically the eyes, nose, and mouth), and the differences between the patterns are relatively subtle in terms of the spatial distribution of fixations [16,23]. The findings from the EMHMM literature demonstrate the importance of integrating transition information into analyses, to detect more nuanced differences between individuals.

Despite recent efforts to better characterise individual differences in eye movement patterns during face recognition using hidden Markov models [15,16,17], the existing literature is limited by its exclusive use of static facial stimuli. There are several reasons that necessitate extension of this early work to dynamic (i.e., moving) facial stimuli, not least because the faces we engage with in everyday life move. They move both rigidly (e.g., head nodding and shaking) and non-rigidly (elastic facial motion, e.g., expressions, speech), and these facial movements carry a substantial amount of socially important information (e.g., speech, emotional state, and potentially intentions and goals). A true understanding of individual differences in eye movements during face recognition requires that our conclusions extend to ecologically valid stimuli.

Aside from ecological validity, we also cannot assume the findings observed for static faces using hidden Markov models [16,23] can be generalised to moving faces, because there is evidence of differential processing of static and moving faces. Behavioural differences have been found when learning and recognising moving compared to static faces (for review, see [24]). Seeing a face move leads to better learning of previously unfamiliar faces [25,26,27], more accurate and faster face matching [28], and better identification of degraded familiar faces [29,30]. This enhanced recognition performance for moving faces is termed the “*motion advantage*” and has been observed in children as young as 3–4 months old [31] and in individuals with developmental prosopagnosia during familiar face recognition [32] and face matching [33].

These behavioural findings have led to debate about what causes the motion advantage, with the dominant theories being that (a) facial motion facilitates the perception of the three-dimensional structure of a face (the *representation enhancement hypothesis*) and (b) facial motion acts as an additional cue to identity, as we represent characteristic facial motions of individual faces in addition to the invariant structure of the face (the *supplemental information hypothesis*) [34,35]. Consequently, it is theoretically possible that we might observe *qualitatively* different patterns of eye movements for moving compared to static faces—for example, eye movement patterns that are optimal for extracting 3D shape information or those that focus on areas of the face that feature more idiosyncratic movement.

However, other perceptual mechanisms may underpin performance differences for moving compared to static faces, with recent research focusing on whether facial motion alters reliance on holistic and/or featural processing. It has been argued that facial motion might promote more part-based (as opposed to holistic) face processing (see [24] for a review). Indeed, Xiao et al. [36] found that facial movements facilitate part-based, not holistic, face processing in children, adolescents, and adults. However, others argue that holistic processing is a fundamental aspect of face perception that applies equally to both static and moving faces [37,38,39], a finding replicated in both same- and other-race faces [40]. In fact, Lian et al. [40] found that rigid facial motion improves holistic processing of own-race faces during encoding. Whilst the impact of facial motion on holistic/featural face processing continues to be debated, these findings raise the possibility of a *quantitative* difference in eye movement strategies for moving and static faces—in other words, a shift in the degree of holistic and featural processing that might be observable in eye movement patterns.

There is some, though limited, existing research on the effect of facial motion on eye movement patterns during face recognition tasks [24,41]. Xiao et al. [41] examined the role of facial movement in face recognition at the ages of 3, 6, and 9 months, finding that across the age groups infants fixated mostly on the centre of static faces, but with increased age, they fixated longer on the mouth of moving than static faces and less on the eyes of moving than static faces. More recently, Butcher et al. [42] studied the effect of facial movement on adult eye movement patterns during both familiar face recognition and unfamiliar face learning. Participants directed a higher proportion of fixations to the internal features (i.e., eyes, nose, and mouth) of moving faces versus static, and the proportion of fixations to the internal non-feature area (i.e., cheeks, forehead, chin) and external area was significantly reduced for moving compared with static faces. The findings of Butcher et al. [42] suggest that facial motion is associated with increased attention to internal facial features. Collectively, these findings reveal eye movement differences for static and moving faces. However, Butcher et al.’s eye movement analyses did not reveal a significant association with recognition performance in a face learning task; thus, the relationship between eye movements and individual differences in moving faces remains unclear.

The existing work on eye movement differences for static and moving faces uses a traditional approach to studying eye movements with pre-determined ROIs, with a focus on presentation mode differences (static vs. moving) rather than individual differences. As noted above, these analyses carry several limitations, such as averaging out individual variation in eye movements, relying on experimenter-defined ROIs, and not incorporating information about eye movement durations and transitions into the analyses. Due to these limitations, previous studies have been somewhat limited in their ability to identify qualitative differences in processing—for example, shifts from holistic to more analytic processing styles (which, as outlined above, are often most apparent in transition information) or the use of novel eye movement patterns for moving faces (e.g., patterns that may reflect structural encoding or the use of supplemental information). Consequently, examining eye movement patterns, such as those identified in EMHMM analyses, may reveal further differences (or similarities) in how people look at moving and static faces and how these differences are related to face recognition performance. For example, participants might change their eye movement patterns from one strategy to another (e.g., from more analytic/eye-focused to more holistic/nose-focused) when learning and recognising static or moving faces (a quantitative shift), or the EMHMM analysis may reveal different, unique eye movement strategies when learning faces in motion compared to static images (a qualitative change). Further, it may be that these eye movement patterns (which incorporate both duration and order of fixations as well as their location) can account for variability in moving face recognition performance, as has been reported for static face recognition (e.g., [23]).

To date, the only research using the EMHMM approach to compare eye movement patterns in moving and static faces has focused on emotion recognition tasks [21,43] rather than face recognition. Analogous to analyses involving face recognition, these studies revealed two or three general eye movement patterns, which can broadly be construed as centrally focused (or holistic) and more eye-focused (or analytic). Paparelli et al. [43] and Zheng and Hsaio [21] found that the majority of participants adopted a similar eye movement strategy across conditions, regardless of whether the images were moving or static. However, unlike the face recognition literature, there was no clear benefit of one strategy over another [43]. While these studies provide important information about eye movement strategies in moving and static emotion processing, it is difficult to judge how well their conclusions will translate to an identity-based task: emotion recognition and identity are distinct processes (see [44] for a review) relying on information from different facial regions [45], and the EMHMM-based eye movement patterns in the emotion recognition tasks were less well-defined than in the identity-based research (e.g., ROIs tended to be larger and with higher levels of overlap).

To better understand how eye movement patterns differ for moving and static faces in an identity-based task and how this relates to face recognition performance, we applied the EMHMM approach to some of the data from Butcher et al. [42]. Our aims were to (1) examine eye movement patterns when learning faces as static or dynamic images and when recognising faces learnt as static or dynamic images; (2) analyse whether specific eye movement patterns are associated with better face recognition performance; and (3) examine consistency in eye movement patterns for static and dynamic faces. Regarding eye movement patterns, given our previous findings using a pre-defined ROI approach [42], we expected that participants would show qualitatively different patterns of eye movements for dynamic and static faces, with dynamic patterns focused more on the internal face features compared to static patterns, which may show more diffuse and less feature-focused ROIs. In relation to performance, based on previous work applying EMHMM to static face images, we expected that participants adopting a more eye-focused/analytic processing style for static faces would show better performance in static face learning and recognition [16,20,22,23], although it is unclear whether the same relationship would apply to moving face learning and recognition. In terms of consistency, studies applying EMHMMs to emotion recognition data suggest that participants may show minimal eye movement differences between moving and static images [21,43]. However, taking into account the differences between emotion and identity processing [44,45], as well as previous research with moving faces [24,42], it is also feasible that participants may shift to more feature-focused eye movement patterns when learning and recognising moving faces.

## 2. Materials and Methods

The majority of the data presented here were initially reported in Experiment 3 of Butcher et al. [42]. The methods are reported in detail in that paper, but a summary is included below.

### 2.1. Design

Participants completed a face learning experiment, with a repeated-measures design with one independent variable (presentation of the faces during the learning phase: moving or static). For this new analysis, the key dependent variable of interest was the eye movement data (fixation location and duration). Performance on the learning task was also a dependent variable. This was examined using the signal detection theory measure *A*, a bias-free measure of sensitivity that is suitable for non-parametric data [46]. *A* can range from 0 to 1, with *A* = 0.5 representing chance-level performance.

### 2.2. Participants

Eye movement data from 73 participants (M_age_ = 24.74 years, SD = 7.32; 20 males, 52 females, all reporting normal or corrected-to-normal vision) were included in the current analyses. Unlike in Butcher et al. [42], no exclusions were made based on behavioural performance, as we were primarily interested in eye movement patterns, and there were no obvious problems with the eye movement data for any participants in the sample. A post hoc power calculation indicated that this sample size has sufficient power to detect large (f = 0.40; d = 0.80) effects in a one-way ANOVA with three groups. This is in line with effect sizes in previous studies that have examined differences in performance related to eye movement patterns [23].

The data were collected across two sites (Brunel University of London and Teesside University). Participants took part voluntarily or in return for course credit. Informed consent was obtained in writing prior to the beginning of the experiment, and ethical approval was granted at both institutions.

### 2.3. Materials and Apparatus

The learning phase of the experiment included 20 faces from the Amsterdam Dynamic Faces Expression Dataset (ADFES [47]). The moving images showed the face moving from a neutral expression to a joyful expression. The static images showed a single frame from the peak of the expression (smiling). All clips (static and moving) were 4 s long, and all stimuli were 720 × 576 pixels in size and displayed in full colour. Participants viewed half of the faces moving and half of the faces static (counterbalanced between participants).

The test phase included 40 static face images: 20 displaying the same identities as in the learning phase (learnt faces), and 20 displaying new identities (distractor faces). The learnt faces showed the same faces from the ADFES, but this time displaying a single frame from the peak of the angry expression. The 20 distractor faces also displayed an angry expression and were selected from several validated emotional face databases (RADIATE [48], Radboud database [49], KDEF [50]). All test phase stimuli were edited using Adobe Photoshop to replace distinctive clothing and backgrounds with a standard grey overlay and to standardise the lighting and pupil alignment across the faces. All test phase stimuli were 720 × 576 pixels and were displayed in full colour.

The experiment was programmed and presented using SR Research Experiment Builder software version 2.4.1 (SR Research Ltd., Kanata, ON, Canada). For participants at Brunel University of London, eye movements were recorded via an EyeLink 1000 desk-mounted video-based eye tracker, which recorded eye movements at 1000 Hz (SR Research Ltd., Kanata, ON, Canada). Viewing distance was held constant at 60 cm with a chin rest. For participants at Teesside University, eye movements were recorded via an EyeLink II head-mounted, video-based eye tracker (SR Research Ltd., Kanata, ON, Canada), at a sampling rate of 250 Hz. Viewing distance was maintained at 65 cm with a chin rest.

### 2.4. Procedures

Prior to the start of the main experiment, participants carried out a nine-point eye movement calibration and validation procedure within the EyeLink API software version 4.594. They then moved onto the learning phase. In the learning phase, participants were instructed to learn a set of 20 faces (10 moving and 10 static, presented in a randomised order). Each learning trial consisted of a 4 s video/static image of a face. Participants were not required to respond.

After completing the learning phase, participants moved directly onto the test phase. In the test phase, participants viewed 40 faces (20 learnt and 20 distractors, presented in a randomised order) and were asked to indicate whether they had seen each face earlier in the experiment via a keypress. Participants were asked to respond as quickly and as accurately as possible, and the face remained onscreen until a response was recorded. There was no time limit in each trial. At the end of each trial, participants were shown a drift correction fixation. The next trial was triggered manually by the experimenter when the participant focused on the drift correction fixation.

### 2.5. Data Analysis

#### 2.5.1. Behavioural Data

Hits (correct identification of learnt face) and false alarms (incorrectly identifying a distractor face as a learnt face) were combined into the signal detection measure *A* [46], separately for faces learnt moving and static. As distractors were not assigned to either movement category, the false alarm rate was common to both conditions. *A* was selected due to the non-parametric distribution of the hit rate (moving faces) and false alarm rate, and because this allowed clear comparisons with the findings from Butcher et al. [42], who used the same measure.

#### 2.5.2. Eye Movement Data

Eye movement data were analysed using the EMHMM toolbox version 0.80 [15], obtained from http://visal.cs.cityu.edu.hk/research/emhmm/ (accessed on 30 July 2024). The EMHMM toolbox uses hidden Markov models, a form of time series model that can be used to describe a series of observations that depend on unobservable, or “hidden”, states, and where the current state depends only on the previous state (a Markov chain). In this analysis, the observations are the fixation sequences in the eye movement data (i.e., spatial coordinates, duration, and sequence), and the hidden states of the HMM represent eye movement regions of interest with duration (ROIDs). Each ROID is represented as a three-dimensional Gaussian emission, with their dimensions corresponding to the spatial locations (x and y coordinates) and duration of fixations. The Gaussian emission represents the probabilistic association between the observed data (e.g., eye fixation locations and durations) and the hidden state the ROID is modelling. This approach has been applied to model eye movement data during a range of face processing tasks (e.g., identification, emotion processing) and is particularly useful for analysing the effects of different factors on eye movements to faces (e.g., drawing experience [20], cognitive ability [22], own-race biases [23]), and how individual differences in eye movement patterns are associated with performance on various tasks [17,23,43].

We began by splitting each participant’s eye movement data by experiment stage (learning or test) and condition (moving or static—for the test phase, this reflected the condition the faces were learnt in, as all test images were static). We then applied a variational Bayesian expectation maximisation algorithm to each dataset to determine the HMM parameters—this determines the optimal number of hidden states. For these analyses, we set the maximum number of hidden states to four. The prior distributions for the Gaussian emissions were Normal-Wishart distributions, with the centre of the image and population mean fixation duration set as prior means. For the spatial dimensions, we set the prior mean to be the centre of the image; for the temporal dimensions, we set the prior mean and standard deviation to be the mean and standard deviation of the whole sample (these were calculated separately for each analysis). Prior to the analysis, fixations falling to the edges of the screen (outside the face image) were cropped. This resulted in the exclusion of 145 fixations in total across all conditions (141/17,271 or 0.82% of fixations in the learning phase; and 5/7635 or 0.06% of fixations in the test phase). The central area of the screen included in the analysis was 472 × 424 pixels.

Once the individual HMM models had been derived, we used a variational hierarchical expectation maximisation algorithm (VHEM) to cluster the individual HMMs and to generate a representative cluster HMM, displaying the cluster centre and common ROIDs and transitions within each cluster. The number of ROIDs within each cluster was determined by the median number of ROIDs within the individual models entered into the VHEM. For each participant, we calculated the log likelihood of their eye movement patterns belonging to each of the different clusters. This acts as a measure of how similar each participants’ individual eye movements were to each of the representative cluster HMMs. These log likelihood values were used to identify the number of clusters and to measure the relationship between eye movement patterns and performance.

To identify the appropriate number of clusters within the dataset, we initially generated two clusters; then, for each cluster, we compared the log likelihood values for the two representative HMMs (in other words, testing if the eye movements from cluster 1 resemble cluster 1 significantly more than they resemble cluster 2). If the t-tests comparing the mean log likelihoods for the different HMMs were significant, we increased the number of clusters and reran the analyses. If any of the t-tests were not significant, we concluded that the clusters were not significantly different and reverted to the next highest number of clusters.

To measure relationships between eye movement patterns and performance, we correlated behavioural outcomes with the log likelihood values. We also calculated a scale of how much each participant’s eye movements resembled each representative HMM along dimensions, contrasting the different HMMs (here dubbed HMM A and HMM B), using the following calculation:

(LL_A_ − LL_B_)/(|LL_A_| + |LL_B_|), (1)

where LL_A_ and LL_B_ represent the log likelihood of an eye movement pattern belonging to two different representative HMMs. In previous research, this has been referred to as the A-B scale, with more positive values indicating greater similarity to HMM A and a negative value indicating greater similarity to HMM B [21,22]. In the current study, we calculated this for each pairing of HMMs (resulting in up to 3 A-B scales when three clusters were identified).

In the first round of analysis, we applied the above clustering method separately for each condition (moving and static) and each experiment stage (learning and recognition)—i.e., all of the HMMs from the learning stage with moving faces were clustered into groups; then, all of the HMMs from the learning stage with static faces were clustered into groups separately; this was repeated for the test stage. These analyses were used to explore and compare eye movement patterns for moving and static faces (and faces learnt in movement or from static images) during the identification task as well as to examine how these eye movement patterns were related to accuracy in the task. As the focus was on the effect of movement, we did not include the distractor faces from the test phase (which were only ever seen as static images) in the analyses. Subsequently, we clustered participants’ HMMs from the moving and static conditions together (although we still analysed the learning and test phases separately) to examine whether participants showed similar eye movements across movement conditions.

## 3. Results

### 3.1. Learning Phase

**Figure 1:**
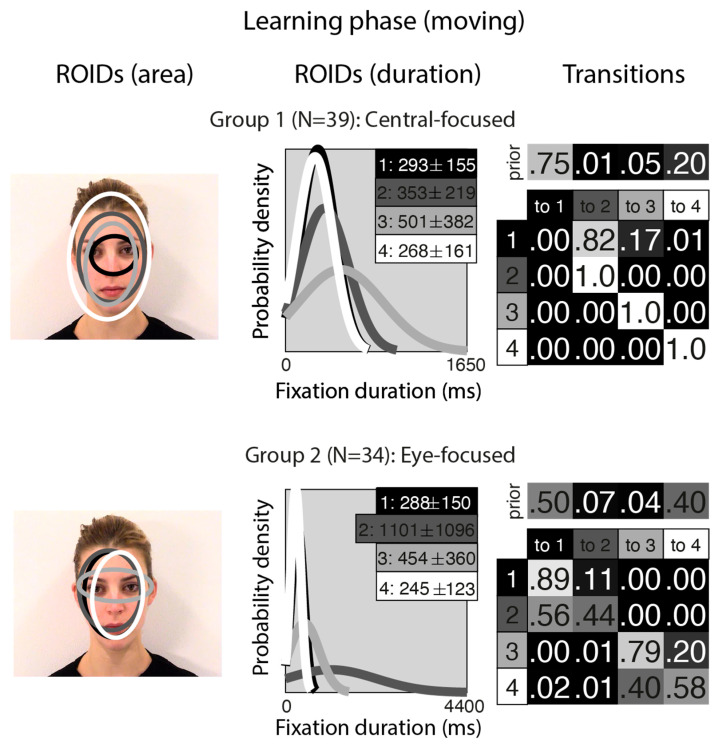
Eye movement patterns for moving faces in the learning phase. The ROIDs (area) column shows the centre and two standard deviations around the centre of each ROID. The ROIDs (duration) column shows the distribution of fixation durations (in ms) within each ROID; the boxes at the top right of each duration distribution figure display the mean and standard deviation of the fixations in each ROID (M ± SD). For both groups, ROIDs 1 and 4 have very similar duration distributions, and the curves overlap. The Transitions column shows the prior probability of fixation patterns beginning in each ROID (top row) and the probability of transitions between ROIDs (bottom matrix). The colour of the ROID spatial and temporal distributions corresponds to the colour of the background of the boxes showing M ± SD and in the transition table. Black: ROID 1; Dark grey: ROID 2; Light grey: ROID 3; White: ROID 4.

Figure 1 shows the groups obtained when clustering the HMMs from viewing moving in the learning stage. For moving faces, our analyses indicated that two clusters were sufficient to summarise the data. The median number of ROIDs from the individual HMMs in the analysis was four; thus, each representative cluster HMM included four ROIDs. In the first group (henceforth *Central-focused*), containing 39 participants, all four ROIDs were centred around the middle of the face, with the smallest ROID (1: black) focused around the nose bridge. Although several of the ROIDs show substantial spatial overlap (e.g., 2 and 3), in some cases they can be distinguished by the length of fixations directed to them (see *ROIDS (duration)* panels in Figure 1). Likewise, the distribution of fixation durations for some ROIDs is practically indistinguishable (e.g., 1 and 4), but the spatial distribution of fixations may differ (see *ROIDs (area)* panels in Figure 1).

People in the Central-focused group typically (around 61% of the time) started with a relatively short fixation (M = 293 ms) in this central ROID before transitioning to another relatively short fixation (M = 353 ms) to an area encompassing the internal facial features—the wider face area (2: dark grey), or less commonly (around 13% of the time), a longer (M = 501 ms) fixation to a similar area (3: light grey). Less frequently (around 20% of the time), participants made repeated fixations within ROID 4 (white), which encompasses the entire face area.

In the second group (henceforth *Eye-focused*), containing 34 participants, the smallest ROID (3: light grey) was focused around the eyes, and the others were centred around the middle of the face. Once again, there was some overlap between ROIDS (specifically, 1 and 2), but these can be distinguished by the length of fixations directed to them (see *ROIDS (duration)* panels in Figure 1). Participants in this group typically (around 90% of the time) started with a short fixation (M = 245–288 ms) to an area encompassing the facial features (ROIDs 1: black and 4: white, which show substantial overlap spatially and temporally and can be collapsed together). Their next fixation tended to stay in this inner facial area (around 52% of the time), although it sometimes (around 20% of the time) moved to a longer fixation (M = 454 ms) to the eye region (3: light grey).

**Figure 2:**
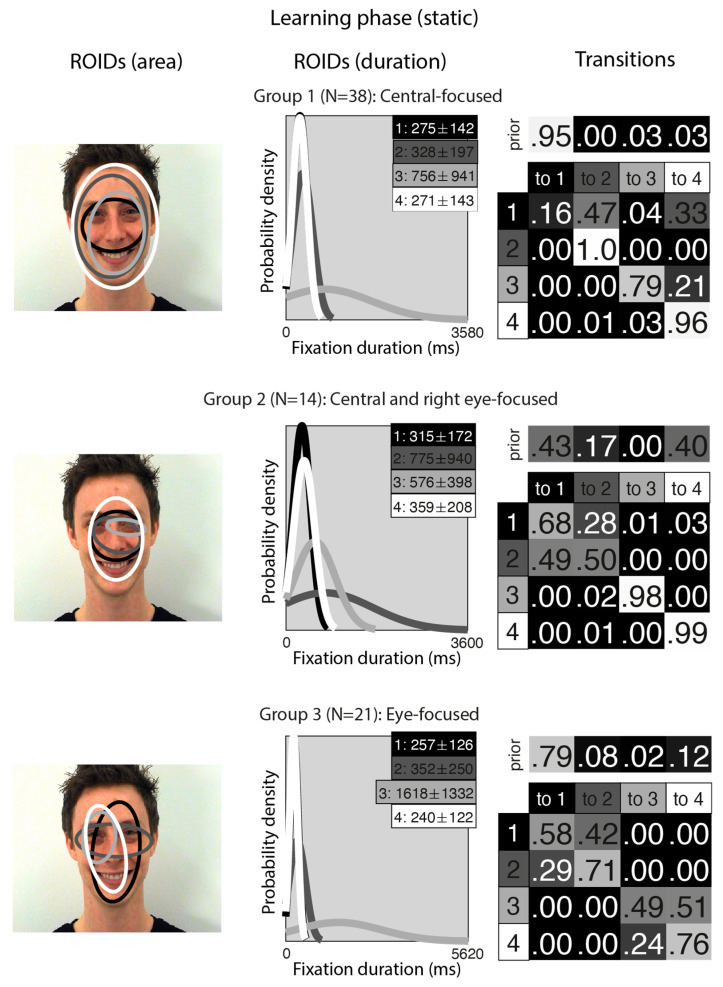
Eye movement patterns for static faces in the learning phase. The ROIDs (area) column shows the centre and two standard deviations around the centre of each ROID. The ROIDs (duration) column shows the distribution of fixation durations (in ms) within each ROID; the boxes at the top right of each duration distribution figure display the mean and standard deviation of the fixations in each ROID (M ± SD). For groups 1 and 3, ROIDs 1 and 4 have very similar duration distributions, and the curves overlap. The Transitions column shows the prior probability of fixation patterns beginning in each ROID (top row) and the probability of transitions between ROIDs (bottom matrix). The colour of the ROID spatial and temporal distributions corresponds to the colour of the background of the boxes showing M ± SD and in the transition table: Black: ROID 1; Dark grey: ROID 2; Light grey: ROID 3; White: ROID 4.

Figure 2 shows the groups obtained when clustering the HMMs from viewing static in the learning stage. For static faces, the data could be clustered into three significantly different groups. The median number of ROIDs from the individual HMMs in the analysis was four, so each representative cluster HMM included four ROIDs. 

The first group, with 38 participants, was very similar to the *Central-focused* cluster from the moving faces, but with an even stronger tendency to begin by with a short fixation (M = 275 ms) around the nose bridge (1: black), before moving to a region encompassing the entire face (ROIDs 2: dark grey and 4: white) around 74% of the time. The second group, with 14 participants, resembled the first, Central-focused group, except the broadest ROID (4: white) was more focused on the internal facial features (as opposed to the entire face in the Central-focused group), and there was one ROID focused around the right eye specifically. Participants in this group tended to begin with a relatively short fixation in a ROID centred on the internal facial features (1: black or 4: white), then either repeatedly fixated within the same ROID (around 60% of the time) or, less commonly, transitioned to a long (M = 775 ms) fixation centred around the bridge of the nose (2: dark grey) (around 14% of the time). We refer to this cluster as the *Central and right eye-focused* cluster.

The third group, with 21 participants, was very similar to the *Eye-focused* cluster from the moving faces, although ROID 4 (white) was more lateralised to the left. Once again, participants typically started with a short fixation (M = 257 ms) to an area encompassing the facial features (1: black); from here, they often remained in the same ROID for several fixations (around 27% of the time) or transitioned directly to a relatively short fixation (M = 352) to the ROID focused on the eye region (2: dark grey) (around 30% of the time). Less commonly, participants in this cluster transitioned from a short fixation (M = 240 ms) to a left-biased region of the face (4: white) to a long fixation (M = 1618) on the left eye (3: light grey).

As we were interested in the relationship between eye movement patterns and accuracy, we (1) ran two one-way ANOVAs examining whether individuals in different eye movement groups performed differently in the behavioural task; and (2) examined the correlations between performance and the log likelihood of individuals belonging to each group. Three extreme outliers in the behavioural data were removed prior to the analyses.

*A* for moving trials did not differ significantly between the Central-focused group (M = 0.85, SD = 0.09) and the Eye-focused group (M = 0.84, SD = 0.10) identified in the moving faces’ EMHMM, F(1,68) = 0.33, *p* = 0.565, η^2^p = 0.00. There was no significant correlation between moving *A* values and the log likelihood of participants belonging to the Central-focused (*r* = 0.06, *p* = 0.600) and Eye-focused (*r* = 0.02, *p* = 0.858) eye movement patterns and no significant correlation between *A* and the A-B scale when contrasting Central-focused and Eye-focused HMMs (*r* = 0.08, *p* = 0.527).

Likewise, *A* for static trials did not differ significantly between the three groups (Central-focused: M = 0.84, SD = 0.83; Eye-focused: M = 0.81, SD = 0.78; Central and right eye-focused: M = 0.78, SD = 0.10; F(2,67) = 2.09, *p* = 0.131, η^2^p = 0.06). There was no significant correlation between static *A* values and the log likelihood of participants belonging to the Central-focused (*r* = −0.07, *p* = 0.569) or Eye-focused (*r* = 0.12, *p* = 0.324), or Central and right eye-focused (*r* = −0.18, *p* = 0.144) eye movement patterns. *A* values did not correlate with the A-B scale contrasting Central-focused and Central right eye-focused groups (*r* = 0.19, *p* = 0.114), but there was a significant correlation between *A* for static trials and the A-B scales when contrasting the Eye-focused pattern with the Central-focused (*r* = −0.25, *p* = 0.034) and the Central right eye-focused pattern (*r* = −0.35, *p* = 0.003). In both cases, a more negative A-B scale value, indicative of more Eye-focused eye movement patterns, was associated with better identification performance.

In sum, analyses of the learning phase revealed two similar clusters in the moving and static conditions: one centrally focused and one more eye-focused. These may be broadly in line with the holistic and analytic patterns reported previously using the EMHMM technique [16,22,23]. The analysis also revealed a third cluster of eye movements for static faces (central and right-eye focused), which was not present for moving faces. There was a tendency for people whose eye movement patterns were more aligned with eye-focused compared to central-focused (or central and right eye-focused) to show better performance in the static condition. However no other associations between eye movement and performance were significant.

### 3.2. Test Phase

**Figure 3:**
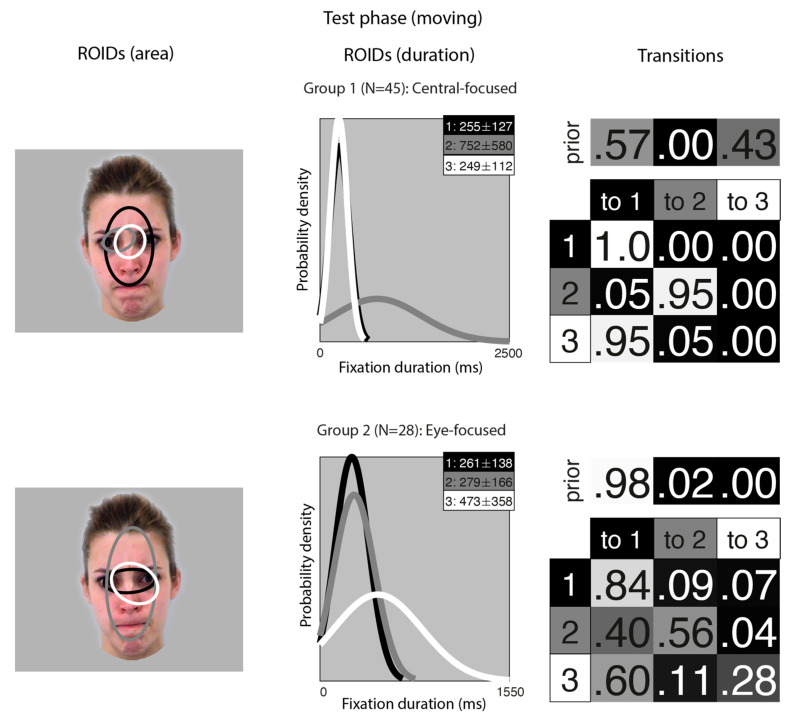
Eye movement patterns for faces learnt in motion, when viewed in the test phase. The ROIDs (area) column shows the centre and two standard deviations around the centre of each ROID. The ROIDs (duration) column shows the distribution of fixation durations (in ms) within each ROID; the boxes at the top right of each duration distribution figure display the mean and standard deviation of the fixations in each ROID (M ± SD). For group 1, ROIDs 1 and 3 have very similar duration distributions, and the curves overlap. The Transitions column shows the prior probability of fixation patterns beginning in each ROID (top row) and the probability of transitions between ROIDs (bottom matrix). The colour of the ROID spatial and temporal distributions corresponds to the colour of the background of the boxes showing M ± SD and in the transition table. Black: ROID 1; Light grey: ROID 2; White: ROID 3.

Figure 3 shows the groups obtained when clustering the HMMs from viewing moving faces in the test (recognition) phase. The median number of ROIDs from the individual HMMs was three; thus, the representative cluster HMMs for moving faces included three ROIDs. As in the learning phase, there was significant spatial overlap between several of the ROIDs, but these could be distinguished by the fixation durations within them.

For faces learnt in motion, two clusters were sufficient to summarise the data. In the first group, containing 45 participants, two ROIDs were focused on the centre of the face, containing short fixations (M ~ 250 ms) encompassing the entire nose (1: black) or just the top of the nose (3: white). The third ROID (2: dark grey) contained longer fixations (M = 752 ms) to the left eye only. The majority of sequences in this group (57%) consisted of repeated short fixations in the larger ROID (3: black), although a significant proportion (41%) started in the smaller, nose-focused ROID (1: white) and moved to the larger ROID. We refer to this as the *Central-focused* group.

In the second group, containing 28 participants, all ROIDs were centred around the top of the nose. The larger ROID (2: dark grey) extended to cover the forehead and mouth region; a smaller ROID containing longer fixations (M = 473 ms) was centred on the top of the nose and encompassed the right eye (3: white); and the final ROID covered the top of the nose and some of the eyes. Typically, participants in this group began with a short fixation (M = 261 ms) to the top of the noise/eye region (1: black), and most fixation sequences in this group (70%) were repeated short fixations to this small area. We refer to this pattern as the *Eye-focused* group.

**Figure 4:**
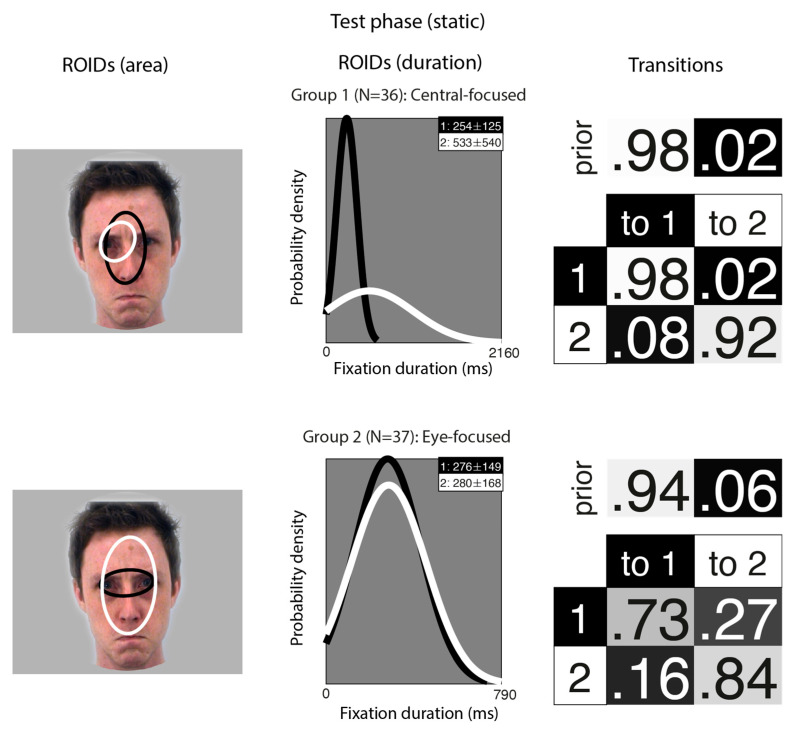
Eye movement patterns for faces learnt as static images, when viewed in the test phase. The ROIDs (area) column shows the centre and two standard deviations around the centre of each ROID. The ROIDs (duration) column shows the distribution of fixation durations (in ms) within each ROID; the boxes at the top right of each duration distribution figure display the mean and standard deviation of the fixations in each ROID (M ± SD). The Transitions column shows the prior probability of fixation patterns beginning in each ROID (top row) and the probability of transitions between ROIDs (bottom matrix). The colour of the ROID spatial and temporal distributions corresponds to the colour of the background of the boxes showing M ± SD and in the transition table. Black: ROID 1; White: ROID 2.

Figure 4 shows the groups obtained when clustering the HMMs from viewing static faces in the test phase. The median number of ROIDs from the individual HMMs was two; thus, the representative cluster HMMs for static faces included two ROIDs. For faces learnt as static images, two clusters were sufficient to summarise the data. Each cluster contained only two ROIDs, both located fairly centrally on the face. In the first group (36 participants), one ROID was characterised by short fixations (M = 254 ms) to an area encompassing the nose (1: black); the other was characterised by longer fixations (M = 533 ms) to the left eye (2: white). Participants in this group typically made repeated short fixations to the nose ROID (1: black), with 95% of the sequences in this group falling into this pattern. While the focus was more limited than in other groups, this group still showed a broad resemblance to the *Central-focused* patterns in other analyses.

In the second group (37 participants), one ROID was characterised by fixations to the nose and eye region (1: white), while the other focused only on the eyes (2: white); both typically included short fixations (M ~ 280 ms). However, unlike the first group, most fixation sequences in this group began in the smaller eye ROID (1: black), and the majority of sequences in this group (51%) consisted of repeated fixations to this ROID. However, it was somewhat more common for participants in this group to transition from the eye ROID to the broader features ROID (2: white) (16%); whereas, in the first group, there were very few transitions from the nose to the eyes (8%). This group resembles the *Eye-focused* groups from other analyses.

Analyses to investigate the relationship between these eye movement patterns and accuracy found no difference between Central-focused and Eye-focused groups in performance on the task for faces learnt in motion, F(1,68) = 0.21, *p* = 0.647, η^2^p = 0.00, or faces learnt as static images, F(1,68) = 0.12, *p* = 0.728, η^2^p = 0.00. *A* for faces learnt in motion did not correlate with the log likelihood for Central-focused eye movement patterns (*r* = 0.18, *p* = 0.140) or Eye-focused eye movement patterns (*r* = 0.08, *p* = 0.487). There was no significant correlation between moving *A* and the A-B scale when contrasting Central-focused and Eye-focused HMMs (*r* = 0.07, *p* = 0.579). Likewise, there were no significant correlations between *A* for faces learnt as static images and the log likelihood for Central-focused eye movement patterns (*r* = 0.18, *p* = 0.138) or Eye-focused eye movement patterns (*r* = −0.01, *p* = 0.967) or the A-B scale when contrasting Central-focused and Eye-focused HMMs (*r* = 0.09, *p* = 0.434).

In sum, analyses of the test phase revealed two patterns of eye movements, broadly similar to the central- and eye-focused patterns in the learning phase. These patterns were very similar for faces learnt in motion and those learnt as static images. This suggests that there is limited carry-over of any motion-related differences in eye movements from learning to recognition.

### 3.3. Consistency in Eye Movements

Next, we entered all the data from the learning phase into one analysis (although we still generated separate individual HMMs for each participant in the moving and static conditions; in essence, this meant each participant contributed two datapoints to the cluster analysis). This allowed us to visualise overall patterns of eye movements that emerged across conditions and to examine whether participants’ eye movements from each condition tended to be clustered together. In short, this analysis allowed us to examine whether people used different eye movement strategies when viewing faces in motion and static images.

**Figure 5:**
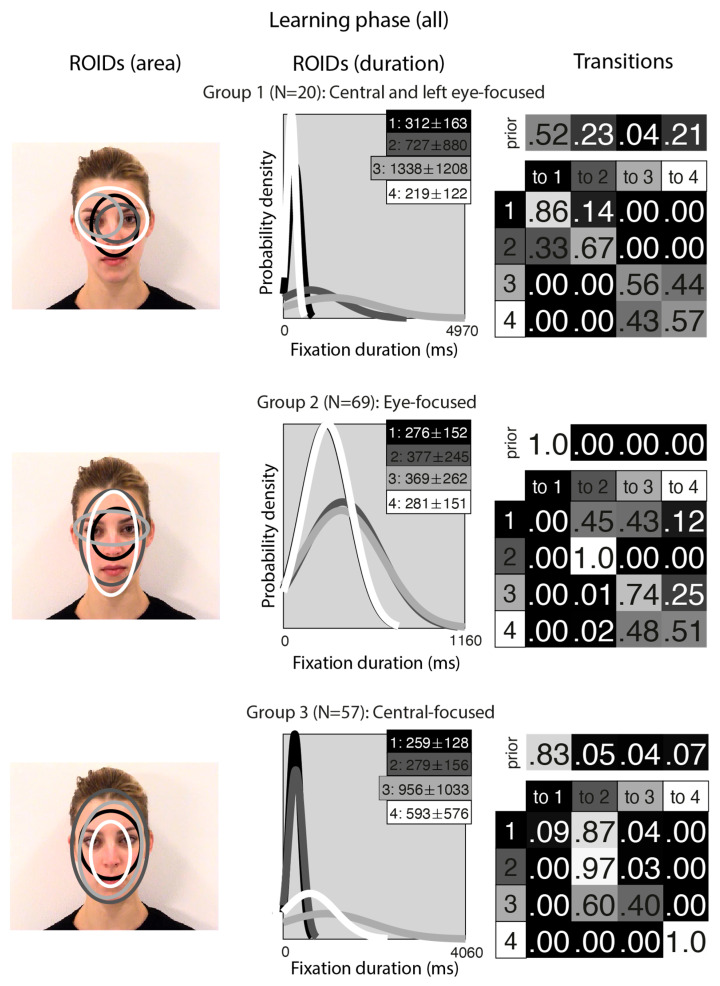
Eye movement patterns for all faces in the learning phase. The ROIDs (area) column shows the centre and two standard deviations around the centre of each ROID. The ROIDs (duration) column shows the distribution of fixation durations (in ms) within each ROID; the boxes at the top right of each duration distribution figure display the mean and standard deviation of the fixations in each ROID (M ± SD). For groups 1 and 2, ROIDs 1 and 4 have very similar duration distributions, and the curves overlap. The Transitions column shows the prior probability of fixation patterns beginning in each ROID (top row) and the probability of transitions between ROIDs (bottom matrix). The colour of the ROID spatial and temporal distributions corresponds to the colour of the background of the boxes showing M ± SD and in the transition table. Black: ROID 1; Dark grey: ROID 2; Light grey: ROID 3; White: ROID 4.

Unsurprisingly, the clusters that emerged from the analysis of the learning stage (Figure 5) closely resembled those from the separate analyses of the learning stage conditions. In the *Central-focused* group (Group 3, 57 datapoints), all four ROIDs were focused around the centre of the face, and participants tended to direct most fixations to the ROIDs that encompassed the inner features (1: black) or the entire face (2: dark grey). In the *Eye-focused* group (Group 2, 69 datapoints), participants tended to begin by fixating on the upper nose and eye region and frequently transitioned from there to the eye region. The final group, which we call *Central and left eye-focused* (Group 1, 20 datapoints), resembled the Central and right eye-focused group from the initial analysis of static faces, but with an ROID centred on the left eye instead of the right eye.

The frequency of participants being allocated to each cluster is displayed in Table 1. Out of 73 participants, 53 (72.6%) showed consistent eye movement patterns across the moving and static faces.

**Figure 6:**
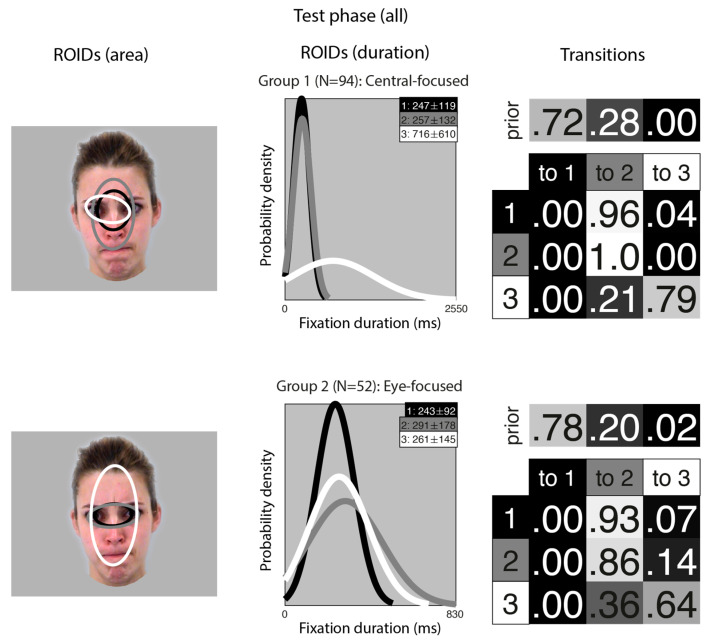
Eye movement patterns for all learnt faces in the test phase. The ROIDs (area) column shows the centre and two standard deviations around the centre of each ROID. The ROIDs (duration) column shows the distribution of fixation durations (in ms) within each ROID; the boxes at the top right of each duration distribution figure display the mean and standard deviation of the fixations in each ROID (M ± SD). The Transitions column shows the prior probability of fixation patterns beginning in each ROID (top row) and the probability of transitions between ROIDs (bottom matrix). The colour of the ROID spatial and temporal distributions corresponds to the colour of the background of the boxes showing M ± SD and in the transition table. Black: ROID 1; Light grey: ROID 2; White: ROID 3.

A similar analysis for the test phase (learnt items only) resulted in two clusters, each with three ROIDs that covered similar areas (Figure 6). However, as in the main analysis, the transitions differentiated the groups.

In the Central-focused group (Group 1, 94 datapoints), most sequences started in a small central ROID (1: black) and then transitioned to a broader ROID covering most of the internal facial features (3: grey). In the Eye-focused group (Group 2, 52 datapoints), most sequences included repeated fixations to a small ROID encompassing the eye region (1: black, and 2: grey). 

The frequency of participants being allocated to each cluster is displayed in Table 2. Out of 73 participants, 61 (83.6%) showed consistent eye movement patterns across moving and static faces.

Overall, there is little evidence that people change their eye movement pattern when learning moving vs. static faces; or when recognising faces learnt in motion or as static images.

## 4. Discussion

This study aimed to examine eye movement patterns when learning moving compared to static faces, using an EMHMM analytic approach that has, until now, only been applied to static face recognition. The analysis revealed three key findings. First, we identified common eye movement patterns for the moving and static faces (in separate and combined analyses), with similar patterns found regardless of whether the face was seen moving or static. Second, we found that the majority of individuals used consistent eye movement strategies for moving and static face learning (learning phase analyses) as well as recognition of faces learnt as moving and static images (test phase analyses). That is, participants’ individual eye movement patterns did not switch between eye-focused and central-focused, depending on whether the face was seen moving or static. Instead, if an individual used an eye-focused pattern when learning and recognising static faces, they typically remained eye-focused when learning and recognising moving faces too; this was the same for those in the central-focused group. Third, we found limited evidence that eye movement patterns (at learning or testing) are related to task performance. The only exception to this was the finding that individuals whose eye movement patterns were more aligned with eye-focused compared to central-focused (or central and right eye-focused) during face learning show better performance in the static condition. These key findings will now be explored in more detail.

This is the first EMHMM analysis to our knowledge that examines the effect of facial movement in a face learning task. The results, in both the learning and test phases, are consistent with previous work that proposed more holistic vs. more analytic eye movement patterns are engaged during face identification (analogous to the central-focused and eye-focused HMMs in the current study) [15,16,20,22]. The condition-specific EMHMM analyses showed relatively similar patterns of eye movements for moving and static faces. In the learning phase, both static and moving eye movement patterns were clustered into broadly similar clusters: Central-focused, where all ROIDs were focused around the centre of the face (in each case, this cluster included roughly half of the participants), and Eye-focused, which included an eye-specific ROIDs alongside other centrally focused ROIDs. These are roughly analogous to the holistic and analytical patterns reported in previous face recognition studies using only static faces [15,16,22]. One notable exception to the consistency across conditions is that the analysis also revealed a third cluster of eye movement patterns for static faces, which was not present for moving faces (the Central and right eye-focused pattern). The third group also appeared in the analysis of the learning phase overall. This group appeared to be a blend of the other two, with an overall focus on central ROIDs but including a lateralised eye-focused ROID.

In theory, the presence of the third group might be seen as evidence for qualitatively or quantitatively different eye movement patterns for moving and static faces. However, there are a number of caveats to this conclusion. First, this group was relatively small compared to the other groups so should be interpreted with some caution. Second, the pattern of eye movements in the third group does not appear to align with predictions of more holistic processing in static compared to moving faces [36]; nor does the difference between the moving and static eye movement patterns resemble the more internal-feature-focused pattern we might expect based on our ROI-based analyses of the same data [42]. Finally, this pattern of eye movements is not unique to studies that use moving faces: other studies using EMHMM analyses have occasionally identified three groups [23,43], with the third group including an ROI with a stronger focus on a single eye or side of the face, as in the current study. Consequently, it is feasible that the third cluster reflects a more lateralised pattern of eye movements in a minority of people (as opposed to more spatially balanced typical analytical strategies, such as our Eye-focused group). The lateralisation of gaze patterns has been associated with perceiver and face gender [51], suggesting that the presence of this pattern may be associated with sample and/or stimulus characteristics rather than the presence of facial movement.

In the recognition phase, the EMHMM analysis revealed two eye movement patterns, which were again relatively similar regardless of whether the faces were learnt in motion or as static images. Like in the learning phase, the two patterns broadly distinguished between participants who focused more on the central areas of the face and those who focused more on the eye region. The consistency of these patterns across different conditions and studies suggests that individuals adopt a limited number of visual strategies during face learning and recognition and that these strategies are relatively robust to differences in stimulus format (the current study) and factors such as age [17,22] and culture [23]. While both strategies take in information from areas of the face that are computationally “optimal” for face processing tasks [4], it is currently unclear whether these visual strategies are driven by innate mechanisms or shaped by experience with faces during development.

Future work may consider how these eye movement patterns relate to other individual differences in eye movements that have been reported in the literature. For example, Peterson and Eckstein [8] used an analytical approach that focused on identifying individuals’ preferred location for their first fixation to a face and found substantial variability between individuals—for example, some participants fixated higher on the face (eye-lookers), whereas others tended to fixate lower (nose-lookers). It is possible that EMHMM analysis taps into related idiosyncrasies in eye movements (e.g., eye-focused patterns might belong to eye-lookers; nose-focused patterns may belong to nose-lookers); alternatively, the two analyses may be measuring different sources of variation between individuals. Combining the two strategies could provide a more thorough characterization of visual strategies during face learning.

Given the similarity of patterns across the moving and static conditions, it is relatively unsurprising that participants showed relatively consistent eye movements when viewing moving and static faces: only a minority of people (16–27%) changed their eye movement patterns from one group to another as a result of movement. The similarity of patterns and high degree of consistency across conditions is in line with studies on emotional face processing, which find that people adopt relatively consistent patterns of eye movements for dynamic and static faces during emotion recognition tasks [21,43]. However, in the context of a face identification task, the lack of distinction between moving and static faces (especially in the learning phase) is somewhat unexpected, especially as previous ROI-based work with the same data has indicated significant differences between moving and static faces [42]. On the other hand, it is relatively unsurprising that we find similar patterns of eye movements for faces learnt in motion or as static images in the test phase, given the test images were all static. Despite reporting a significant motion advantage (that is, better face recognition performance for faces learnt as moving videos than as static images), traditional ROI-based analyses on the same dataset also found no significant differences in the test phase between faces learnt as moving and static images [42]. Taken together, these findings suggest that the eye-movement-based effects of learning a face in motion are relatively small. Further, they suggest that the EMHMM-based findings in the literature, which are based on analyses of static images (e.g., the relationship between eye movement patterns and different person characteristics [20,22,52]), likely generalise to more ecologically valid moving faces as well. However, future work may wish to extend this technique to examine eye movement patterns for moving and static familiar faces. Previous behavioural work suggests that the motion advantage in face recognition might be more prominent for familiar than unfamiliar faces [34], so it is possible the effects of movement on eye movements are more pronounced for famous or familiar faces.

Our findings suggest relatively small differences in eye movement patterns for moving and static faces. However, it is highly likely that eye movement patterns may vary based on other factors, such as task demands. Previous work examining face learning and recognition found that about 40% of participants changed their eye movement patterns between learning and testing [16]—far higher than the level of variability we observed when comparing moving and static images. While we cannot directly compare across the learning and test phases, due to different numbers of groups in each stage (3 for learning and 2 for test), our results suggest that a relatively similar number of participants adopted central- (39.0%) and eye-focused (47.2%) patterns in the learning phase, while the majority of participants (64.3%) adopted the eye-focused pattern in the test phase. This suggests some degree of variability between the learning and test phases, potentially due to the differing demands of initial encoding and subsequent recognition. Broadly, these differences align with the idea that gaze follows functions [53]—that is, eye movement patterns are driven not just by stimulus characteristics but also by the demands of the task at hand.

One notable difference between moving and static faces was their association with performance. There were very few significant associations between eye movement patterns and face recognition performance in the task. Eye movement patterns were not associated with performance for moving faces in the learning phase or for any faces in the test phase. Likewise, group-based analyses and simple log likelihood measures were not associated with performance for static faces in the learning phase. However, there was a tendency for people whose patterns were more aligned with eye-focused compared to central-focused (or central and right eye-focused) in the learning phase to show better performance in the static condition. This finding aligns with previous work [16,20,22,23] and adds to a growing body of evidence for an association between analytic/eye-focused eye movement patterns and static face recognition. However, our results suggest that the same associations do not apply to performance with moving faces. Currently, we cannot account for this difference.

One simple explanation for the absence of an association between eye movements and moving face recognition performance is power. While our sample size is equivalent to many previous studies using this analytical approach, the introduction of movement might increase the heterogeneity of eye movements and therefore weaken the relationship with performance. It is also possible that the task or stimuli were too simple, which could have restricted the range of performance and limited the potential associations that could be detected. However, given the presence of a significant association for static faces and eye-focused movement patterns, these explanations seem unlikely. Similarly, the fact that we found a significant behavioural motion advantage, and that Butcher et al. [42] reported significant differences in eye movements between moving and static faces, suggests that the limited difference between patterns of eye movements in the two conditions cannot be dismissed as an artefact of the task or stimuli.

Alternatively, it may be that recognition for moving faces is driven at least partially by factors that we did not measure, such as attention. We note that one prominent hypothesis related to the movement advantage (the social signals hypothesis) suggests that movement may increase attention to the face, thus improving face encoding [54]. It is therefore possible that the presence of movement resulted in higher engagement with the stimuli, leading to better performance without substantially altering eye movement patterns. Further work is needed to replicate and extend these findings to different forms of facial motion (e.g., social movements as in the current study, compared to non-social movements such as chewing), as this may shed light on the role of social cues in driving these effects. On the other hand, it may be that people adopt idiosyncratic, performance-maximising eye movement patterns, akin to the idiosyncratic performance-maximising first fixations reported by Peterson and Eckstein [8], and that these are particularly well-adapted to moving faces (potentially due to their ecological validity). If this is the case, the specific eye movement pattern is of less relevance to performance than the alignment between the individual and the eye movement pattern. Paradigms that restrict viewing or limit the use of typical eye movements during the recognition of moving faces (e.g., [1,55]), could address this point and shed further light on the functionality of eye movements during face learning and recognition.

To our knowledge, this is one of the first times that both EMHMM and ROI-based analyses have been applied to the same dataset (however, see [52]) and thus one of the first opportunities to compare the findings from these two analytical approaches. The contrast in findings for the learning phase between Butcher et al. [42] and the current findings might reflect the different strengths and focuses of the two analytical approaches: a traditional ROI-based approach may be more likely to detect quantitative and/or more spatially-based changes, i.e., an increased focus on specific areas of the face, as we found in [42], while the EMHMM analyses may pick up more qualitative differences in eye movements strategies (e.g., analytic vs. holistic, or variation in how people transition between ROIs). It is possible these qualitative differences are more likely to be observed when comparing across participants, as opposed to conditions. For example, differences in eye movement strategies have often been linked with characteristics of the perceiver, such as age and cognitive decline [22], portraiture experience [20], social anxiety [52], and strength of holistic processing [56]. On the other hand, EMHMM analyses generally report similar eye movement patterns despite variations in face stimuli. For example, participants exhibit similar eye movement patterns for faces displaying different emotions [21,43], dynamic and static emotional faces [21,43], and faces of difference races [23]. This supports arguments proposing that individuals show observer-specific consistent idiosyncratic eye movement preferences, which reflect their optimal strategies for face processing within a given task [8]. It is important to note that we are not suggesting that one form of analysis is superior to the other; rather, the analyses may address complementary research questions which, when taken together, can contribute to a more robust understanding of how different factors affect eye movements during face recognition. In line with this idea, the only other paper that has applied both ROI and EMHMM analyses to the same dataset [52] found significant effects for social anxiety using EMHMM analyses, though these effects were not apparent in ROI-based analyses—essentially, the reverse pattern to what we observe in this paper and as observed by Butcher et al. [42]. This supports our point that each analysis has its strengths and limitations and that both can contribute to our understanding of eye movements in face recognition.

In sum, our research indicates that eye movement patterns are relatively stable during face recognition, regardless of whether the faces are learnt in motion or as static images. This has both theoretical and applied implications. Understanding how different patterns of eye movements are associated with face recognition performance and how different factors affect (or do not affect) this relationship can provide more information about the perceptual underpinnings of face recognition in the typical population. For example, our findings do not support the idea that the motion advantage in face recognition is driven by specific patterns of eye movements. However, this knowledge is also important because it can inform future research on face recognition deficits and contribute to the development and evaluation of training programmes and interventions for people who struggle with face recognition (e.g., those with acquired and developmental prosopagnosia) [57,58]. For example, our study adds to a larger body of work suggesting that analytic processing is associated with better face recognition performance. To date, eye movement research in prosopagnosia has relied on traditional ROI-based analyses, but future work may consider applying EMHMM analyses to determine whether individuals with face recognition deficits show similar patterns of eye movements as those with typical face recognition, and if not, whether more optimal patterns of eye movements can be trained.

## 5. Conclusions

In conclusion, this paper used a novel analytical technique (EMHMM) to examine patterns of eye movements when learning and recognising moving and static faces. Broadly, participants’ eye movements could be clustered into two or three groups, representing a divide between more centrally focused and more eye-focused patterns. Participants tended to be fairly consistent in their eye movements toward moving and static faces, and toward faces learnt as moving and static images, and there were limited (small, condition-specific) associations between performance and eye movements. Our findings contrast with previous analyses of the same data but align with other research suggesting consistency of eye movement patterns across various stimulus-related factors, both within and across participants. This highlights the importance of diverse analytical approaches to understanding stimulus-driven, task-based, and individual differences in eye movements.

## Figures and Tables

**Table 1:** Number of participants within each eye movement group in the learning phase, displayed separately for moving and static faces.

		Static Faces			
		Central and Eye	Eye	Central	Total
**Moving faces**	Central- and eye-focused	9	-	-	**9**
	Eye-focused	1	25	12	**38**
	Central-focused	1	6	19	**26**
	**Total**	**11**	**31**	**31**	**73**

Numbers in bold indicate the total number of participants in each group.

**Table 2:** Number of Participants Within Each Eye Movement Group in the Test Phase, Displayed Separately for Faces Learnt as Moving and Static Images.

		Static Faces		
		Eye	Central	Total
**Moving faces**	Eye-focused	20	5	**25**
	Central-focused	7	41	**48**
	**Total**	**27**	**46**	**73**

Numbers in bold indicate the total number of participants in each group.

## Data Availability

The individual participant data on which study conclusions are based and a list of all the stimulus materials have been made publicly available on the Open Science Framework (see https://osf.io/xz2hr/).

## Abbreviations

The following abbreviations are used in this manuscript:

EMHMM	Eye movement hidden Markov model
ROID	Region of interest with duration
HMM	Hidden Markov model
VHEM	Variational hierarchical expectation maximisation
